# LOX inhibition downregulates MMP-2 and MMP-9 in gastric cancer tissues and cells

**DOI:** 10.7150/jca.33223

**Published:** 2019-10-20

**Authors:** Lei Zhao, Haiya Niu, Yutao Liu, Lei Wang, Ning Zhang, Gaiqiang Zhang, Rongqing Liu, Mei Han

**Affiliations:** 1 Department of Pathogenic Biology and Immunology, School of Basic Medical Sciences, Ningxia Medical University;; 2 Department of Rheumatology and Immunology, The General Hospital of Ningxia Medical University, Yinchuan, Ningxia 750004, P.R. China;

**Keywords:** lysyl oxidase, matrix metalloproteinase-2, matrix metalloproteinase-9, β-aminopropionitrile, gastric cancer, PDGF-PDGFR

## Abstract

**Objective:** The objective of this study was to analyze the effects of lysyl oxidase (LOX) on the expression and enzyme activity of the matrix metalloproteinases 2 (MMP-2) and 9 (MMP-9) and to study its preliminary effect mechanisms.

**Methods:** We collected fresh cancer specimens from 49 gastric cancer patients who underwent surgery. Immunohistochemistry was used to quantitate the protein expression levels of LOX and MMP-9 in gastric cancer tissues and to analyze their correlation. Also, six-week old nude mice were divided into a control group and a LOX inhibition group. SGC-7901 gastric cancer cells were inoculated subcutaneously into the backs of the two groups of these mice to construct a gastric cancer-bearing nude mouse model. In the LOX inhibition group, β-aminopropionitrile (BAPN) was used to inhibit LOX. Western blotting was used to quantitate the relative expression levels of MMP-2 and MMP-9 in mouse tumor tissues, and gelatin zymography was used to quantitate their enzyme activity levels. In addition, BGC-823 gastric cancer cells were cultured, then 0.1 mM, 0.2 mM, and 0.3 mM BAPN and 2.5 nM, 5 nM, and 10 nM LOX were added to treat BGC-823 cells. ELISA and gelatin zymography were used to quantitate the protein concentrations and changes in enzyme activity of MMP-2 and MMP-9 in the culture supernatant. Western blotting was used to quantitate the relative expression levels of platelet derived growth factor receptor (PDGFR) in the BGC-823 gastric cancer cells after LOX inhibition and exogenous LOX addition.

**Results:** In the tissues from the gastric cancer patients, the relative expression levels of LOX and MMP-9 were positively correlated (r = 0.326, P < 0.05). Compared with the control group, the tumor tissues from mice in the LOX inhibition group had reduced relative expression levels and enzyme activities of MMP-2 and MMP-9 (P < 0.05). After LOX were inhibited with different concentrations of BAPN in BGC-823 gastric cancer cells, the protein concentrations and enzyme activity levels of MMP-2 and MMP-9 in the culture supernatants were decreased (P < 0.05). In addition, the relative expression level of PDGFR in gastric cancer was decreased when BAPN concentrations increased, showing a negative dose-dependent manner (r_PDGFR-α_ = -0.964, r_PDGFR-β_ = -0.988, P < 0.05). After exogenous LOX treating BGC-823 cells, the concentrations and enzyme activity levels of MMP-2 and MMP-9 in the cell supernatant were increased (P < 0.05). Further, the relative expression of PDGFR in gastric cancer cells was increased with the increase of exogenous LOX, showing a positive dose-dependent manner (r_PDGFR-α_=0.952, r_PDGFR-β_=0.953, P<0.05).

**Conclusions:** LOX inhibition can inhibit the expression and enzyme activity of MMP-2 and MMP-9 in gastric cancer tissues and cells, and the probable mechanism is through its effects on the PDGF-PDGFR signaling pathway.

## Introduction

Malignant tumors are major diseases that severely threaten the health of mankind. Gastric cancer is one of the malignant tumors and is the third leading cause of cancer deaths after lung cancer and liver cancer^1^. Invasion and metastasis are basic biological hallmarks of malignant tumors, and the extracellular matrix (ECM) plays an important role in the invasion and metastasis of tumors^2^, ^3^. Therefore, any substance that can disrupt the dynamic equilibrium of the ECM may be intimately associated with tumor invasion and metastasis^4^.

LOX is an extracellular, copper-dependent monoamine oxidase which acts on the collagen and elastic fiber proteins in the ECM. LOX can catalyze the oxidative deamination of peptidyl lysine and hydroxylysine moieties on the lysine residues of collagen and elastic fibers to form peptidyl aldehydes, ultimately forming covalent crosslinks between collagen and elastic fibers. This plays an important role in the development and maturation of the ECM and the maintenance of ECM stability^5^, ^6^. Changes in LOX expression levels are observed in many human cancers, such as breast cancer, colon cancer, pancreatic cancer, and other cancers^7^, ^8^. LOX is highly expressed in gastric cancer cells with high invasiveness and is intimately associated with distal metastases in gastric cancer^9^. In addition, LOX is an important prognostic factor for gastric cancer^10^.

Matrix metalloproteinases (MMPs) are zinc- and calcium-dependent proteases. Their main functions are to degrade ECM components, promote angiogenesis, and regulate cell-cell adhesion. MMPs have been found to be associated with many types of cancers, such as lung cancer, breast cancer, and colon cancer 11, 12. MMPs and LOX play an important role in ECM remodeling and resynthesis 13.

LOX can aid in establishing the ECM, while MMPs can degrade ECM components. On the surface, it appears that these two proteins have antagonistic biological functions. However, our previous study found that expression of LOX and MMP-2 are positively correlated in gastric cancer tissues, and they play synergistic roles in promoting the invasion and metastasis of gastric cancer^14^. Another of our previous studies, on rheumatoid arthritis, found that inhibition of LOX expression in rat synovial fluid can downregulate MMP-2 and MMP-9 expression^15^. The relationship between LOX and MMP-2 and MMP-9 in tumorigenesis and tumor progression are still unclear.

The platelet derived growth factor receptor is a transmembrane tyrosine kinase receptor that is made up of two subunits (α and β). PDGFR is the major component of the PDGF-PDGFR signaling pathway. It participates in cell proliferation, transformation, invasion, and angiogenesis. The PDGF-PDGFR signaling pathway is intimately associated with tumorigenesis, development, invasion, metastasis, and angiogenesis in gastric cancer^16^, colorectal cancer^17^, and breast cancer^18^, ^19^. Guo et al.^20^ found that the PDGF-PDGFR signaling pathway can induce the epithelial-mesenchymal transition and promote metastasis in gastric cancer by downregulating the expression of E-cadherin. Wang et al.^21^ found that lipoteichoic acid can upregulate MMP-9 expression through the PDGF-PDGFR pathway and increase astrocyte migration in rat brains. Dong et al.^22^ carried out a study on atherosclerosis and found that blocking the PDGF-PDGFR signaling pathway with the tyrosine kinase inhibitor, AG1296, can reduce MMP-2 and MMP-9 expression in atherosclerotic plaques in mice. Our group has previously found that LOX regulates MMP-2 and MMP-9. However, further verification is required to see if the PDGF-PDGFR signaling pathway is involved.

In order to understand gastric cancer further, we investigated the effects of LOX on MMP-2 and MMP-9 and its mechanisms in this disease. We collected fresh cancer specimens from gastric cancer patients who underwent surgery to quantitate the protein expression levels of LOX and MMP-9 in gastric cancer tissues. We constructed a gastric cancer-bearing nude mouse model and used the LOX inhibitor β-aminopropionitrile to inhibit LOX. After LOX inhibition, we analyzed the effects on protein expression and enzyme activity of MMP-2 and MMP-9 in tumor tissues from the nude mouse model. Simultaneously, we examined the expressions and activities of MMP-2 and MMP-9 after LOX inhibition and exogenous LOX treatment in gastric cancer cells, and analyzed the effect of LOX inhibition on PDGFR in the PDGF-PDGFR signaling pathway in gastric cancer cells.

## 1. Materials and methods

### 1.1. Gastric cancer tissues, experimental animals, and cells

This study was approved by the ethics committee of Ningxia Medical University (Yinchuan, China; registration no. 2012-01).

There were 49 gastric cancer patients who underwent surgery at the Department of General Surgery and Department of Tumor Surgery of the General Hospital of Ningxia Medical University. Among these patients, there were 39 males and 10 females, and their mean age was 59.02 ± 11.53 years. All patients signed informed consent. The inclusion criteria were that patients were clinically diagnosed with gastric cancer before operation and patients were confirmed with gastric cancer by histopathological examination after operation. There was no history of other malignant tumors in patients. The exclusion criteria were that patients who received adjuvant therapy such as radiotherapy or chemotherapy before operation. Tumors were staged according to TNM (1997) classification criteria of Union for International Cancer Control (UICC). Patients were divided into T1+T2 group (8cases) and T3 + T4 group (41cases), and were also divided into metastatic group (40cases) and non-metastatic group (9cases) basing on that whether there were local lymph node metastasis and distant metastasis in tumor. Detailed pathological and clinical data were collected for all patients. The cancer tissues were resected from the patients during surgery, then were embedded with paraffin after fixed with 10% formalin.

Nude mice (Nu/nu mice) were purchased from Beijing Vital River Laboratory Animal Technology Co., Ltd. (Approval No.: SCXK (Hu) 2013-0016). The mice were 6 weeks old, weighed 18-22 g, and included both genders. The mice were housed in a specific pathogen-free (SPF) environment. All animal experiments conformed to the institutional animal care and use guidelines.

The SGC-7901 and BGC-823 gastric cancer cell lines originated from lymph node metastases of gastric adenocarcinoma and poorly differentiated gastric adenocarcinoma, respectively, and were purchased from the Shanghai Institutes for Biological Sciences, P.R. China.

### 1.2 Immunohistochemistry

Gastric cancer tissues were sectioned and dewaxed before carrying out antigen retrieval and cooling. This was followed by incubation with 3% H_2_O_2_ to remove endogenous peroxidases. After that, the sections were blocked with 10% donkey serum for 15 minutes, followed by primary antibodies (goat anti-MMP-9 antibody, dilution: 1:20, R&D; rabbit anti-LOX antibody, dilution: 1:100, Abcam) overnight at 4°C. After washing, horseradish peroxidase-labeled anti-goat or anti-rabbit IgG were added as secondary antibodies. This was followed by DAB staining, hematoxylin nuclear staining, dehydration, clearing, and mounting. The sections were observed at 400× magnification using an optical microscope. DPcontroller software was used for photography under the same conditions. At least 5 random high magnification fields were selected for photography for each section. Following that, Image-Pro Plus 6.0 professional image analysis software was used to analyze and quantitate the area of positive expression and integrated optical density (IOD) of all images. The mean optical density (MOD) was used to express the relative expression levels of LOX and MMP-9. MOD = IOD/positive area.

### 1.3 Preparation of the gastric cancer-bearing nude mouse model and treatment

Twenty 6-week old nude mice (nu/nu) were randomized into a control group (n = 10) and a LOX inhibition group (n=10). The mice were fed a standard diet every day and housed under conditions with a light-dark cycle of 12 h/12 h, temperature of 22°C ± 2°C, and humidity of 55% ± 5% for 1 week of acclimatization before the experiments. In the LOX inhibition group, mice were given daily 0.2 mL intraperitoneal injections of the LOX inhibitor, BAPN (Sigma, St. Louis, MO, USA) at a dose of 100 mg/kg. The same volume of sterile PBS was administered via intraperitoneal injections to the control group every day. After 2 weeks, 1×10^7^ SGC-7901 gastric cancer cells/mouse were inoculated subcutaneously into the backs of the two groups of mice to create the gastric cancer-bearing nude mouse model. BAPN or PBS intraperitoneal injections were still administered. After 4 weeks from inoculation of tumor cells, and when model construction was successful, mice from the two groups were anesthetized using ether and euthanized by cervical dislocation. *In situ* tumors were extracted, and total protein was extracted from the mouse xenograft tumors following the manufacturer's instructions in the total protein extract kit (KeyGEN BioTECH Jiangsu, P.R. China). The BCA assay was used for protein quantitation.

### 1.4 Cell culture and treatment

BGC-823 gastric cancer cells were cultured until the logarithmic growth phase. Then, 0.25% trypsin was used to digest the cells to prepare a single cell suspension. The cells were seeded at 10^5^ cells/well in a 24-well plate, and RPMI-1640 culture medium (Gibco, Carlsbad, CA, USA) containing 10% FBS was added. The plates were cultured in a 37°C, 5% CO_2_ incubator. After 24 hours of culture, the culture medium was changed with RPMI-1640 culture medium containing BAPN at different concentrations (0 mM, 0.1 mM, 0.2 mM, 0.3 mM) and LOX at different concentrations (0nM, 2.5nM, 5nM, 10nM). After another 48 hours of culture, the culture supernatant was collected.

### 1.5 Western Blotting

We carried out 10% polyacrylamide gel electrophoresis, for which 30 μg of the specimens were loaded in each well. Following that, the proteins were transferred onto PVDF membranes (EMD Millipore, Billerica, MA, USA). The membranes were blocked with 5% skim milk for 1 hour at room temperature before incubation with primary antibodies (rabbit anti-MMP-2 antibody or rabbit anti-MMP-9 antibody, dilution: 1:300, Santa Cruz; rabbit anti-PDGFR-α antibody or rabbit anti-PDGFR-β antibody, dilution: 1:50, Santa Cruz; rabbit anti-β-actin antibody, dilution: 1:1000, KeyGEN BioTECH Jiangsu, P.R. China) for 3 hours at room temperature. Horseradish peroxidase-labeled anti-rabbit or anti-goat IgG were used as secondary antibodies (dilution: 1:2000). After DAB staining, the membrane was placed in a gel imager for scanning. Quantity One software was used for quantitation. The following equation was used to calculate the relative expression of proteins: relative protein expression = target protein intensity/internal control (β-actin) intensity.

### 1.6 Gelatin zymography

Total protein samples from mouse tumor tissues were mixed with equal volumes of 2× loading buffer (0.125 M Tris-HCl pH 6.8, 4% SDS, 20% glycerol, 0.04% bromophenol blue). The supernatants from gastric cancer cells were mixed with 5× loading buffer (0.4 M Tris-HCl pH 6.8, 10% SDS, 50% glycerol, 0.03% bromophenol blue) in a 4:1 ratio. Electrophoresis was carried out using 8% SDS-PAGE gel (with 1% gelatin in the separation gel, Sigma G-9382), and 10 μg was added per well. After electrophoresis, the gel was placed in 2.5% Triton X-100 (CAS: 9002-93-1, Biotopped, Beijing, China) and shaken at 4°C at a low speed for 3 × 30 min to elute SDS. The gel was then placed in the incubation buffer (50 mM Tris-HCl pH 7.5, 5 mM CaCl_2_, 0.2 M NaCl_2_, 1 μM ZnCl_2_) and incubated at 37°C for 42 hours, and then stained with Coomassie Brilliant Blue R250 for 40 min with slow shaking. The dyed gel was placed in a decolorizing solution (20% methanol, 10% acetic acid) and decolorized by shaking at room temperature, and the decolorizing solution was replaced with fresh solution every 30 min until the transparent bands due to MMP-2 and MMP-9 digestion could be seen within the blue background. The gel was placed in a gel image analysis system (UVPGDS-8000, BIO-RAD) and scanned. The Gel-Pro 4.0 professional image analysis software (Media Cybernetics, Maryland, MD, USA) was used for analysis and quantitation of the positive bands to obtain the IOD values. The IOD values represented MMP-2 and MMP-9 enzyme activity.

### 1.7 ELISA

The culture supernatants were used for testing with ELISA kits (Cloud-Clone Corp., Houston, TX, USA), following the manufacturer's instructions. To plot standard curves and to obtain calculation formulas, the absorbances at 450 nm were used as the y-coordinates, and the concentrations of the standards were used as the x-coordinates. The absorbances of the samples were substituted into the resulting formula to calculate the protein concentrations of MMP-2 and MMP-9 in the cell culture supernatants.

### 1.8 Statistical analysis

The SPSS 22.0 statistical software was used for analysis. Quantitative data was expressed as mean ± standard deviation. Inter-group comparison of quantitative data that conformed to a normal distribution was carried out using the two-sample t-test of the means. The relationship between two variables was analyzed using Pearson's correlation analysis. A difference of P < 0.05 was considered statistically significant.

## 2. Results

### 2.1 Correlation between the relative expression levels of LOX and MMP-9 in cancer tissues from gastric cancer patients

**Figure 1:**
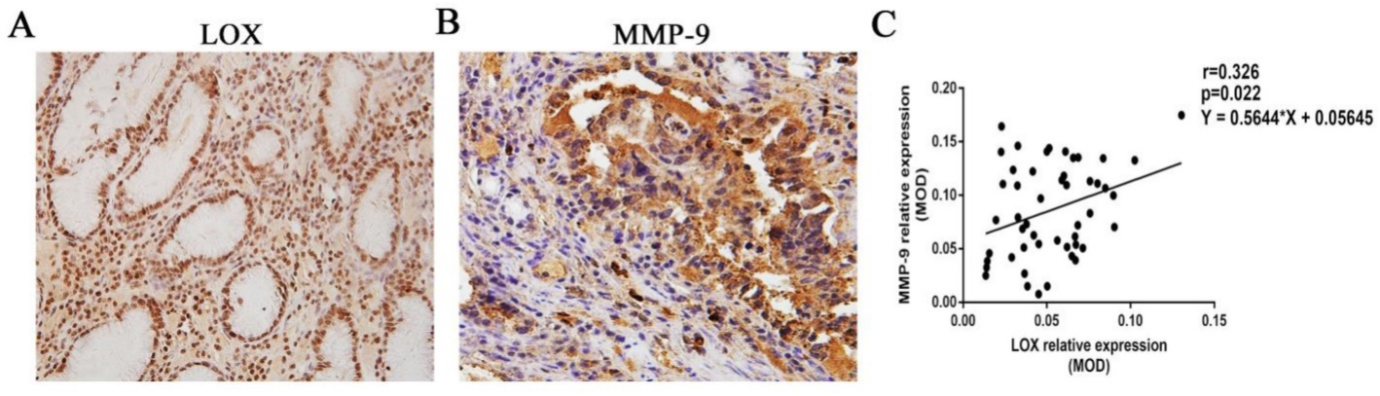
** Correlation between the relative expression of LOX and MMP-9. A, B.** Immunohistochemistry was used to quantitate the relative expression levels of LOX and MMP-9 in the tissues from gastric cancer patients. **C.** It was found that the relative expression levels of LOX and MMP-9 in gastric cancer tissues were positively correlated, r = 0.326, P < 0.05.

The relative expression levels of LOX and MMP-9 were 0.052 ± 0.025 and 0.086 ± 0.043, respectively, in the tissues from the 49 gastric cancer patients. LOX and MMP-9 showed a positive correlation (r = 0.326, P = 0.022) (Fig. 1).

### 2.2 Changes in the relative expression levels and enzyme activity of MMP-2 and MMP-9 in tumor tissues from mice after LOX inhibition

**Figure 2:**
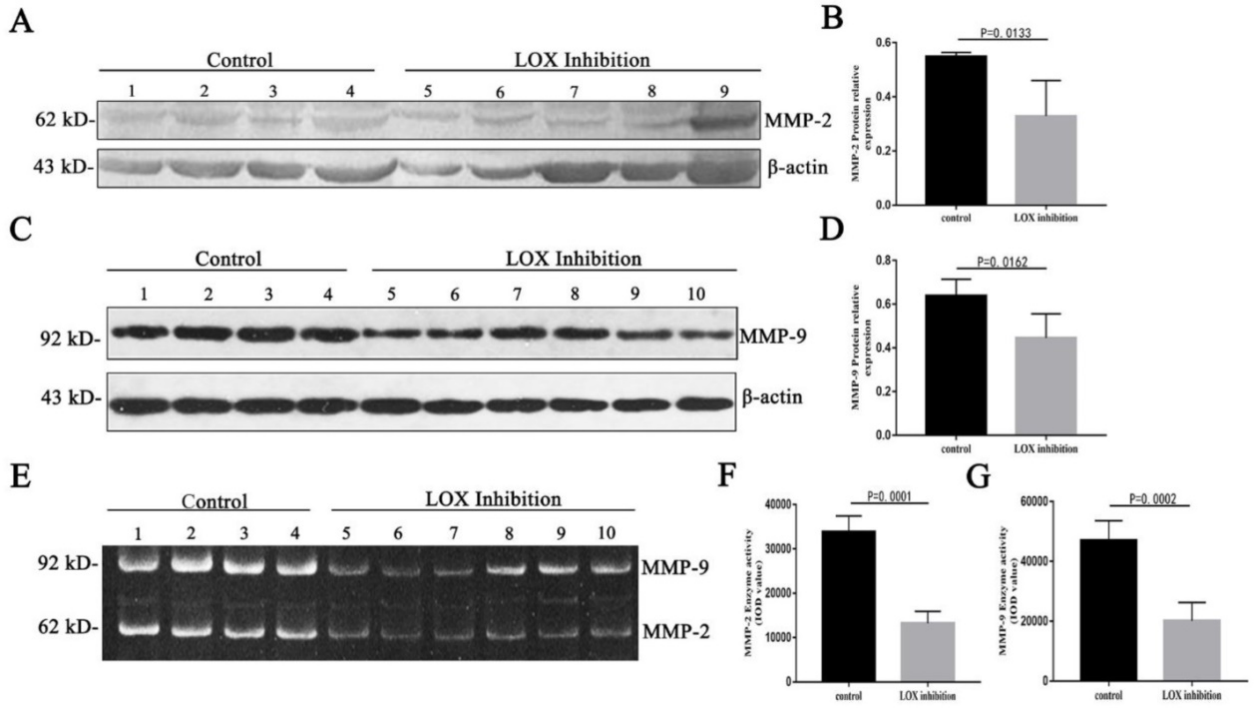
** Changes in relative expression levels and enzyme activity of MMP-2 and MMP-9 in mouse tumor tissues after LOX inhibition. A.** Western blot quantitation of MMP-2 expression levels in mouse tumor tissues. Lanes 1-4: control group; 5-9: LOX inhibition group (intraperitoneal injection of BAPN in mice). **B.** Relative expression levels of MMP-2 in mouse tumor tissues after LOX inhibition were significantly lower than in the control group, P < 0.05. **C.** Western blot quantitation of MMP-9 expression levels in mouse tumor tissues. Lanes 1-4: control group; 5-10: LOX inhibition group. **D.** Relative expression levels of MMP-9 in mouse tumor tissues after LOX inhibition were significantly lower than in the control group, P < 0.05. **E.** Enzyme activity of MMP-2 and MMP-9 in mouse tumor tissues by gelatin zymography. Lanes 1-4: control group; 5-10: LOX inhibition group. **F.** Reduced MMP-2 enzyme activity was seen in mouse tumor tissues when compared with the control group, P < 0.05. **G.** Reduced MMP-9 enzyme activity was seen in mouse tumor tissues when compared with the control group, P < 0.05.

After BAPN was used to inhibit LOX in mouse tumor tissues, the relative expression level of MMP-2 (62 kDa) in the LOX inhibition group was significantly decreased compared with the control group (Table 1, Fig. 2A, 2B) (P < 0.05), and the relative expression level of MMP-9 (92 kDa) in the LOX inhibition group was significantly decreased compared with the control group (Table 1, Fig. 2C, 2D) (P < 0.05). When the LOX inhibition group was compared with the control group, the enzyme activity of the active form of MMP-2 (62 kDa) in mouse tumor tissues was significantly decreased (Table 1, Fig. 2E, 2F) (P < 0.05) and the enzyme activity of the active form of MMP-9 (92 kDa) was significantly decreased (Table 1, Fig. 2E, 2G) (P < 0.05).

### 2.3 Changes in concentrations and enzyme activities of MMP-2 and MMP-9 in the culture supernatants of BGC-823 gastric cancer cells after LOX inhibition

**Figure 3:**
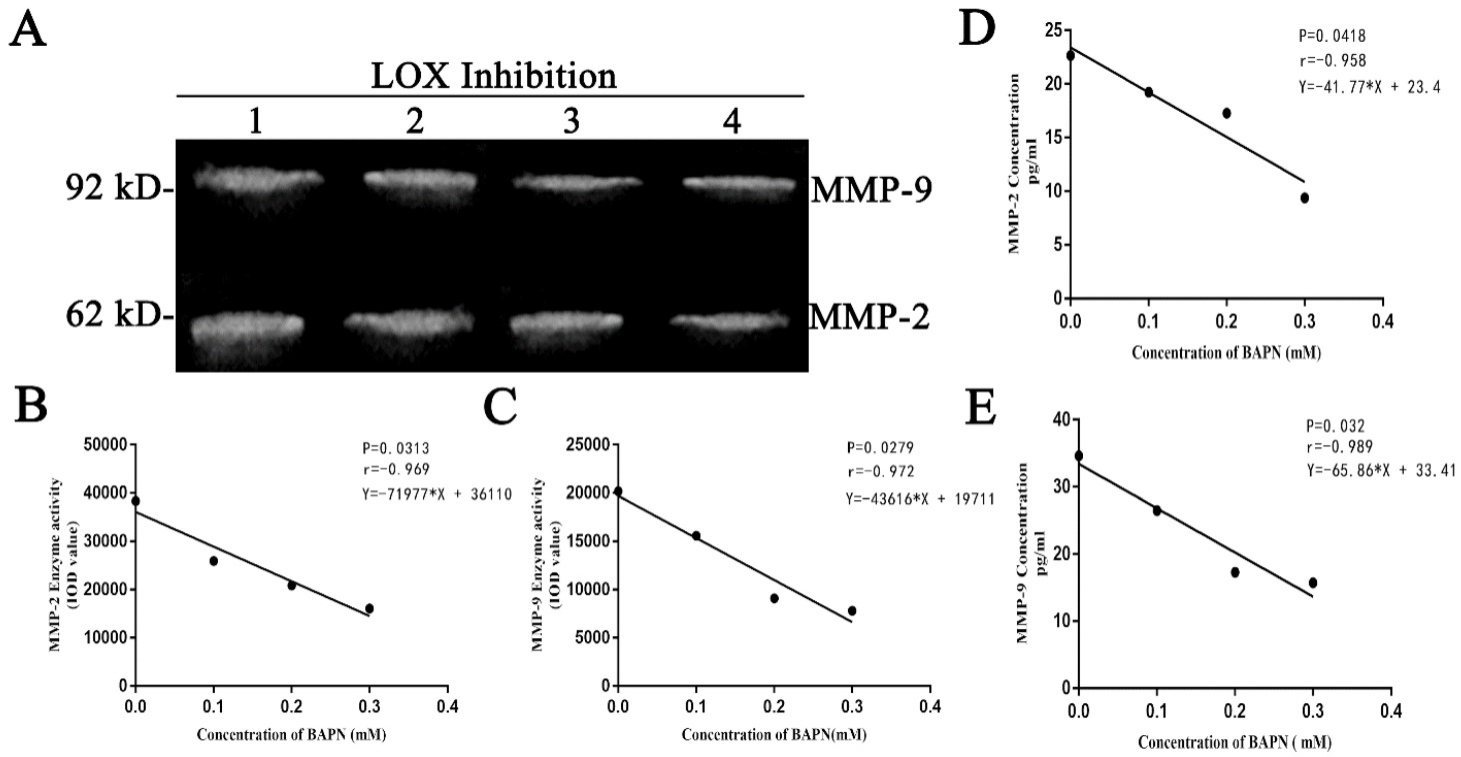
** Changes in concentrations and enzyme activities of MMP-2 and MMP-9 in the cell culture supernatants of BGC-823 cells after LOX inhibition A.** MMP-2 and MMP-9 enzyme activity in cell culture supernatants as quantitated by gelatin zymography, Lanes 1-4: BAPN concentrations of 0, 0.1, 0.2, and 0.3 mM, respectively. The 62 kDa band is MMP-2, and the 92 kDa band is MMP-9. **B, C**. Show MMP-2 and MMP-9 enzyme activities in cell culture supernatants as tested by gelatin zymography. MMP-2 and MMP-9 enzyme activity decreased with increasing BAPN concentration and showed negative correlations, P < 0.05. **D, E.** MMP-2 and MMP-9 concentrations from cell culture supernatants, as quantitated by ELISA after LOX was inhibited in gastric cancer cells by BAPN (0, 0.1, 0.2, or 0.3 mM), showing decreases as BAPN concentrations increased. The protein concentrations of MMP-2 and MMP-9 correlated negatively with BAPN concentrations, P < 0.05.

BAPN was used to inhibit LOX in gastric cancer cells for 48 hours. The enzyme activities of MMP-2 and MMP-9 in the culture supernatants decreased as BAPN concentration increased. BAPN exhibited negative correlations with the enzyme activities of MMP-2 and MMP-9 (Table 2, Fig. 3A, 3B, 3C) (P < 0.05). BAPN (0, 0.1, 0.2, or 0.3 mM) was used to inhibit LOX in gastric cancer cells for 48 hours. The concentrations of MMP-2 and MMP-9 in the culture supernatants decreased as BAPN concentration increased. BAPN exhibited negative correlations with the concentrations of MMP-2 and MMP-9 (Table 2, Fig. 3D, 3E) (p<0.05).

### 2.4 Changes of MMP-2, MMP-9 concentrations and enzyme activities after exogenous addition of LOX in BGC-823 cell

**Figure 4:**
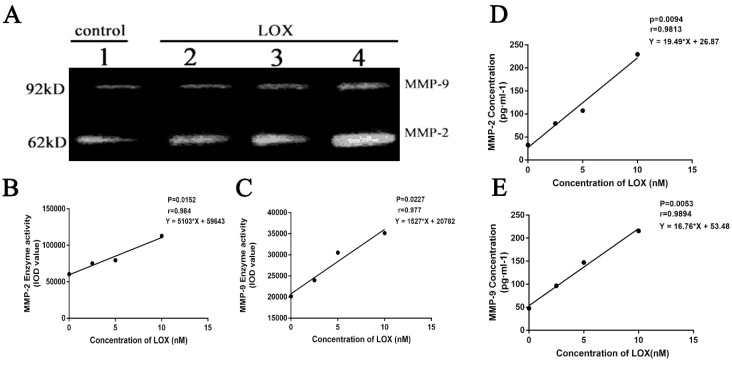
** The effect of exogenous LOX on concentrations and activities of MMP-2 and MMP-9 in BGC-823 cells A.** The activities of MMP-2 and MMP-9 in BGC-823 cell supernatant were detected by gelatin zymography. Lanes 1-4 respectively represent 0, 2.5, 5 and 10 nM LOX. The bands in 62 kDa are for MMP-2, and the bands in 92 kDa are for MMP-9. **B, C.** The activities of MMP-2 and MMP-9 in BGC-823 cell supernatant were increased with the increase of LOX concentration, showing a positive dose-dependent manner (P<0.05).**D, E.** ELISA results showed that the exogenous LOX (0, 2.5, 5, 10 nM) increased the concentrations of MMP-2 and MMP-9 of BGC-823 cell supernatant in a dose-dependent manner (P<0.05).

After BGC-823 cell treated by LOX (0, 2.5, 5 and 10nM) for 48h, the enzyme activities of MMP-2 and MMP-9 were also elevated with the increase of LOX concentration with P < 0.05 (Table 3, Fig. 4A, 4B and 4C), both in a dose-dependent manner. After BGC-823 cell treated by LOX (0, 2.5, 5 and 10nM) for 48h, the levels of MMP-2 and MMP-9 in the cell supernatant were increased with the increase of LOX concentration with P < 0.05 (Table 3, Fig. 4D and 4E).

### 2.5 Change in PDGFR relative expression level in cells after LOX inhibition

**Figure 5:**
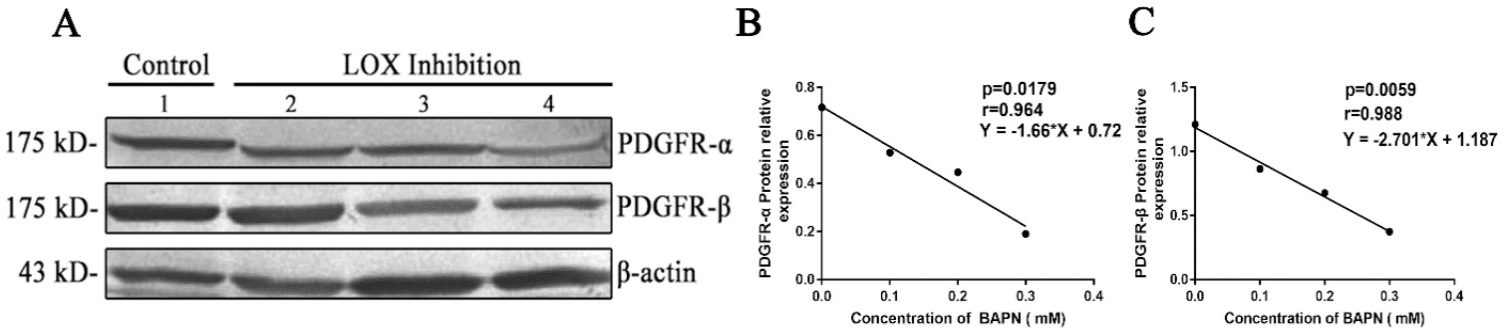
** Effects of LOX inhibition on PDGFR in gastric cancer cells A.** Western blot quantitation of PDGFR-α and PDGFR-β expression in cells. Lane 1: control group, lanes 2-4: LOX inhibition groups (BAPN concentrations of 0.1, 0.2, and 0.3 nM, respectively). **B , C.** As BAPN concentration increases, PDGFR-α and PDGFR-β expression levels in BGC-823 gastric cancer cells decreased and showed negative correlations, P < 0.05.

After different concentrations of BAPN (0, 0.1, 0.2, or 0.3 mM) were added to inhibit LOX in gastric cancer cells for 48h, we found that the relative expression levels of PDGFR-α decreased when BAPN concentrations increased. BAPN concentration showed negative correlations with the relative expression levels of PDGFR-α (Table 4, Fig. 5A, 5B) (P < 0.05). The relative expression levels of PDGFR-β in gastric cancer cells decreased as BAPN concentrations increased. BAPN concentration showed negative correlations with the relative expression levels of PDGFR-β (Table 4, Fig. 5A, 5C) (P < 0.05).

### 2.6 Change of PDGFR relative expression in BGC-823 cell after exogenous addition of LOX

**Figure 6:**
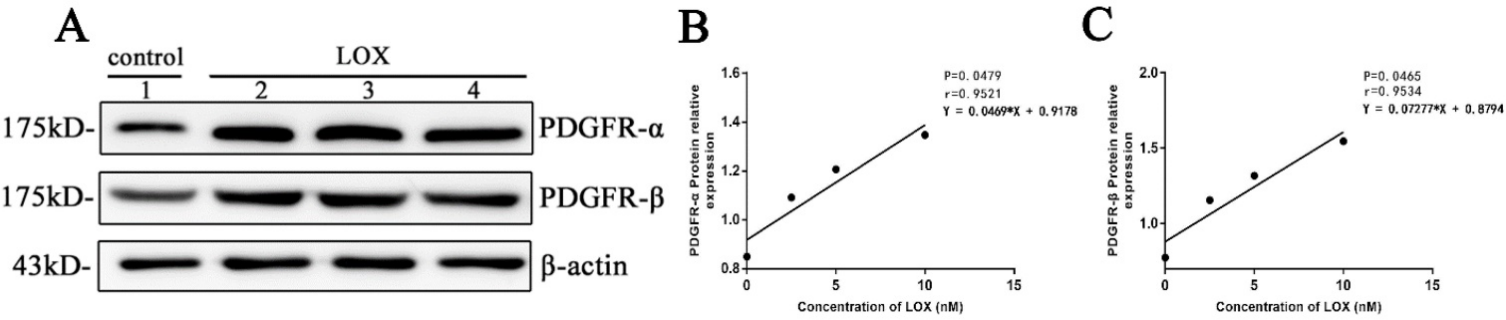
** The effect of exogenous LOX on PDGFR in gastric cancer cell line A.** The expression of PDGFR-α and PDGFR-β in cells was detected by Western-blot. Lane 1: control group. Lanes 2-4: LOX group (The concentrations of LOX respectively at 2.5, 5 and 10 nM). **B.** The expressions of PDGFR-α in gastric cancer cells BGC-823 after treated by exogenous LOX at different concentrations. **C.** The expressions of PDGFR-β in gastric cancer cells BGC-823 after treated by exogenous LOX at different concentrations.

After BGC-823 cell treated by LOX (0, 2.5, 5 and 10nM) for 48h, it was showed that the relative expression of PDGFR-α in cells was increased with the increase of LOX concentration with P < 0.05 (Table 5, Fig. 6A, 6B) and the relative expression of PDGFR-β in cells was increased with the increase of LOX concentration with P < 0.05 in a dose-dependent manner (Table 5, Fig. 6A, 6C).

## 3. Discussion

During tumorigenesis and tumor progression, the ECM maintains the microenvironment for the growth of tumor cells through the dynamic equilibrium of metabolism and turnover. In addition, degradation and remodeling of LOX can promote covalent crosslinking between collagen or elastin in the ECM and maintain ECM stability. This aids in the migration of tumor cells, formation of an invasive phenotype, and vascular intravasation, which are intimately associated with tumor progression and gastric cancer metastasis. Studies have shown that hypoxia can increase LOX expression in gastric cancer cells, promoting epithelial-mesenchymal transition, cell migration, proliferation, and metastasis^23^, ^24^.

MMP-2 and MMP-9 can degrade ECM components, except for polysaccharides. MMP-2 and MMP-9 play an important role in pathophysiological processes such as cell migration, angiogenesis, and the invasion and metastasis of malignant tumors. Yao et al.^25^ found that MMP-2 and MMP-9 overexpression are independent markers for early stage gastric cancer metastasis.

LOX, MMP-2, and MMP-9 all act on the ECM. From their conventional functions, it seems that these proteins are mutually antagonistic to each other. However, our previous research results instead found that LOX and MMP-2 are positively correlated at the mRNA and protein levels in gastric cancer tissues. In gastric cancer tissues with accompanying lymph node metastases, LOX and MMP-2 are positively correlated at the mRNA and protein level. This suggests that LOX and MMP-2 may have synergistic effects during metastasis in gastric cancer^14^. Additionally, our previous study on rheumatoid arthritis also found that LOX expression levels in synovial fluid were positively correlated with the expression levels of MMP-2 and MMP-9. Inhibition of LOX expression in rat synovial fluid can downregulate MMP-2 and MMP-9 expression levels^15^.

In order to confirm the relationship between LOX and MMP-2 and MMP-9 in tumorigenesis and tumor progression, we studied LOX, MMP-2, and MMP-9 in human tumor tissues, animal models, and *in vitro* cell culture.

First, we collected cancer specimens from 49 gastric cancer patients who underwent surgery. Immunohistochemistry was used to quantitate the expression levels of LOX and MMP-9 in gastric cancer tissues. We found that LOX and MMP-9 expression were positively correlated in the gastric cancer tissues of these 49 patients. Our previous study has also demonstrated that LOX and MMP-2 expression are positively correlated in tissues from gastric cancer patients^14^.

Further, we constructed a gastric cancer xenograft model in nude mice. After intraperitoneal injection of these mice with BAPN to inhibit LOX activity, we observed changes in MMP-2 and MMP-9. We found that the expression and enzyme activity levels of MMP-2 and MMP-9 were decreased in mouse tumor tissues after LOX inhibition. Additionally, we found that the concentrations and enzyme activities of MMP-2 and MMP-9 in cell culture supernatant showed dose-dependent decreases after different concentrations of BAPN were added to inhibit LOX in gastric cancer cells, and these decreases were negatively correlated with BAPN concentrations. The results of the animal model experiments and *in vitro* cell experiments demonstrate that LOX inhibition can inhibit the expression and enzyme activity of MMP-2 and MMP-9. This suggests that LOX can upregulate the expression and enzyme activity of MMP-2 and MMP-9, which verified our results in human tumor tissues.

The LOX family has five members including LOX, LOXL, LOXL2, LOXL3 and LOXL4. Studies have shown that BAPN inhibits all these five isoenzymes. However, the expression levels of the five isoenzymes are different in different tissues and cells, in which LOX and LOXL are expressed in most tissues, while the expression of LOXL2, LOXL3 and LOXL4 genes is more restricted^26^. The relative expression of LOX in BGC-823 cells was much higher than that in other members, and the levels and activities of MMP-2 and MMP-9 were significantly increased after exogenous LOX addition. Therefore, it was considered that LOX had a regulatory effect on MMP-2 and MMP-9.

The PDGF-PDGFR signaling pathway is intimately associated with tumorigenesis and tumor progression. PDGFR is highly expressed in many types of tumors, such as gastric cancer and breast cancer, and is associated with the degree of malignancy in tumors^27^, ^28^. PDGFR is composed of two subunits, PDGFR-α and PDGFR-β, which bind different ligands. Akio et al. found that the gene expression levels of PDGFR-β are significantly increased in gastric cancer tissues. That group also found that PDGFR-β is associated with proliferation of gastric cancer cells and is an independent predictive factor for patient survival^29^. Borkham et al. found that the PDGF-PDGFR signaling pathway can upregulate the expression of tissue inhibitor of metalloproteinase-1 and decrease the enzyme activity of MMP-2 and MMP-9^30^. A study on atherosclerosis found that blocking the PDGF-PDGFR signaling pathway can reduce MMP-2 and MMP-9 expression in atherosclerotic plaques in mice^22^. This shows that the PDGF-PDGFR signaling pathway can regulate MMP-2 and MMP-9.

In addition, the study of Lucero et al. showed that LOX can oxidize PDGFR-β on the surface of mouse fibroblasts and promote PDGFR-β synthesis. However, LOX knockout decreases PDGFR-β on the cell surface, significantly decreasing PDGF-PDGFR signal transduction^31^. In this study, we cultured BGC-823 gastric cancer cells and added different concentrations of BAPN to inhibit LOX activity. We found that the expression levels of both PDGFR-α and PDGFR-β were significantly decreased. In addition, BAPN concentration was negatively correlated with the expression of PDGFR-α and PDGFR-β, in agreement with the findings of Lucero et al. When LOX was added to gastric cancer cell line BGC-823, the expression of PDGFR-α and PDGFR-β was increased, and it showed a positive dose-dependent manner. This suggests that LOX can oxidize PDGFR on the cell surface to regulate the PDGF-PDGFR signaling pathway.

In summary, during tumor invasion and metastasis, MMP-2 and MMP-9 are used to degrade the ECM and basement membrane so that tumor cells can detach, invade, and metastasize. At the same time, metastasized and newly formed tumor cells require LOX to construct new ECM and interact with the surrounding environment after they have reached neighboring or distal tissues, so that these cells can colonize these sites. Therefore, LOX, MMP-2, and MMP-9 are highly expressed in many malignant tumors. The results of this study show that LOX can upregulate PDGFR during tumorigenesis and tumor progression to affect the PDGF-PDGFR signaling pathway. This then increases MMP-2 and MMP-9 expression. LOX is simultaneously upregulated or downregulated with MMP-2 and MMP-9 and acts in synergy with these enzymes. However, whether LOX increases PDGFR-α and PDGFR-β through oxidation still requires further research. In addition, in-depth investigation of whether LOX can affect MMP-2 and MMP-9 through other methods is also needed.

## Figures and Tables

**Table 1:** Changes in relative expression levels and enzyme activities of MMP-2 and MMP-9 in mouse tumor tissues after LOX inhibition

Group	n	Protein relative expression			Enzyme activity (IOD value)	
		MMP-2 (62 kD)	MMP-9 (92 kD)		MMP-2 (62 kD)	MMP-9 (92 kD)
Control	10	0.55 ± 0.01	0.65 ± 0.08		3.4×10^4^ ± 1.7×10^3^	4.7×10^4^ ± 3.3×10^3^
LOX inhibition	10	0.35 ± 0.02	0.45 ± 0.10		1.3×10^4^ ± 1.0×10^3^	2.0×10^4^ ± 2.5×10^3^
t		3.289	3.035		10.47	6.596
p		0.0133	0.0162		0.0001	0.0002

**Table 2:** Changes in concentrations and enzyme activities of MMP-2 and MMP-9 in the cell culture supernatants of BGC-823 cells after LOX inhibition.

Concentration of BAPN (mM)	Concentration (pg·ml^-1^)		Enzyme activity (IOD value)	
	MMP-2	MMP-9	MMP-2 (62 kD)	MMP-9 (92 kD)
0	22.65	34.62	3.8×10^4^	2.0×10^4^
0.1	19.24	26.48	2.6×10^4^	1.5×10^4^
0.2	17.28	17.29	2.1×10^4^	9×10^3^
0.3	9.38	15.73	1.6×10^4^	7.8×10^3^
r	-0.958	-0.989	-0.969	-0.972
p	0.0418	0.032	0.0313	0.0279

**Table 3:** Changes in concentrations and enzyme activities of MMP-2 and MMP-9 in the cell culture supernatants of BGC-823 cells after exogenous LOX addition

Concentration of LOX ( nM)	Concentration (pg·ml^-1^)		Enzyme activity (IOD value)	
	MMP-2	MMP-9	MMP-2(62KD)	MMP-9(92KD)
0	32.65	47.62	6×10^4^	2 x10^4^
2.5	79.24	96.48	7.5×10^4^	2.4 x10^4^
5	107.28	147.29	7.9×10^4^	3.1 x10^4^
10	229.38	215.73	1.1 x10^5^	3.6 x10^4^
r-value	0.9813	0.9894	0.984	0.977
p-value	0.0094	0.0053	0.0152	0.0227

**Table 4:** Effects of LOX inhibition by BAPN on PDGFR in gastric cancer cells

Concentration of BAPN (mM)	Protein relative expression	
	PDGFR-α	PDGFR-β
0	0.717	1.212
0.1	0.529	0.864
0.2	0.447	0.677
0.3	0.191	0.374
r	-0.964	-0.988
p	0.0179	0.0059

**Table 5:** Effect of exogenous LOX on PDGFR in gastric cancer cells

Concentration of LOX (nM)	Protein relative expression	
	PDGFR-α	PDGFR-β
0	0.849	0.773
2.5	1.091	1.154
5	1.206	1.317
10	1.346	1.547
r-value	0.9521	0.9534
p-value	0.0479	0.0465

## Abbreviations

LOX: lysyl oxidase
MMP-2: matrix metalloproteinases-2
MMP-9: matrix metalloproteinases-9
BAPN: β-aminopropionitrile
PDGFR: platelet derived growth factor receptor
ECM: extracellular matrix
MMPs: Matrix metalloproteinases
SPF: specific pathogen-free
IOD: integrated optical density
MOD: mean optical density
